# Supplementary material for: Mutant Kras as a Biomarker Plays a Favorable Role in FL118-Induced Apoptosis, Reactive Oxygen Species (ROS) Production and Modulation of Survivin, Mcl-1 and XIAP in Human Bladder Cancer
Source: Cancers (Basel). 2020 Nov 18;12(11):3413. doi: 10.3390/cancers12113413 (PMC7698790; doi:10.3390/cancers12113413)
Supplement: Supplementary file 1 [file cancers-12-03413-s001.zip › cancers-1002631 -suppl-final/cancers-1002631 -suppl-final.docx]

Article

Mutant Kras as a Biomarker Plays a Favorable Role in FL118-Induced Apoptosis, Reactive Oxygen Species (ROS) Production and Modulation of Survivin, Mcl-1 and XIAP in Human Bladder Cancer

Sreevidya Santha, Xiang Ling, Ieman A. M. Aljahdali, Sailee S. Rasam, Xue Wang, Jianqun Liao, Jue Wang, Christos Fountzilas, Qingyong Li, Jun Qu and Fengzhi Li

Supplementary Materials:

**Figure S1.** FL118 inhibition of bladder cancer cell growth/viability in a series of FL118 concentration for 72h treatment.

**Figure S2.** FL118-mediated induction of differential PARP cleavage in bladder cancer cells.

**Figure S3.** FL118-mediated inhibition of survivin, Mcl-1 and/or XIAP in bladder cancer cell lines.

**Figure S4.** Effect of FL118 on AKT and ERK1/2 expression and phosphorylation (activation) in bladder cancer cells.

**Figure S5.** Sensitivity of bladder cancer cells to FL118 is irrelevant to Top1 expression level after FL118 treatment.

**Figure S6.** Role of mutant Kras in FL118-mediated apoptosis and inhibition of antiapoptotic protein expression.

**Figure S7A.** Cytokine and growth factor signaling related proteins.

**Figure S7B.** Immunotherapy related proteins and/or antigens.

**Figure S7C.** Membrane receptor signaling related proteins.

**Figure S7D.** Cellular (non-membrane) signaling proteins.

**Figure S7E.** DNA and chromatin remodeling related proteins.

**Figure S7F.** Transcription factor or co-factor related proteins.

**Figure S7G.** Gene expression and DNA damage related proteins.


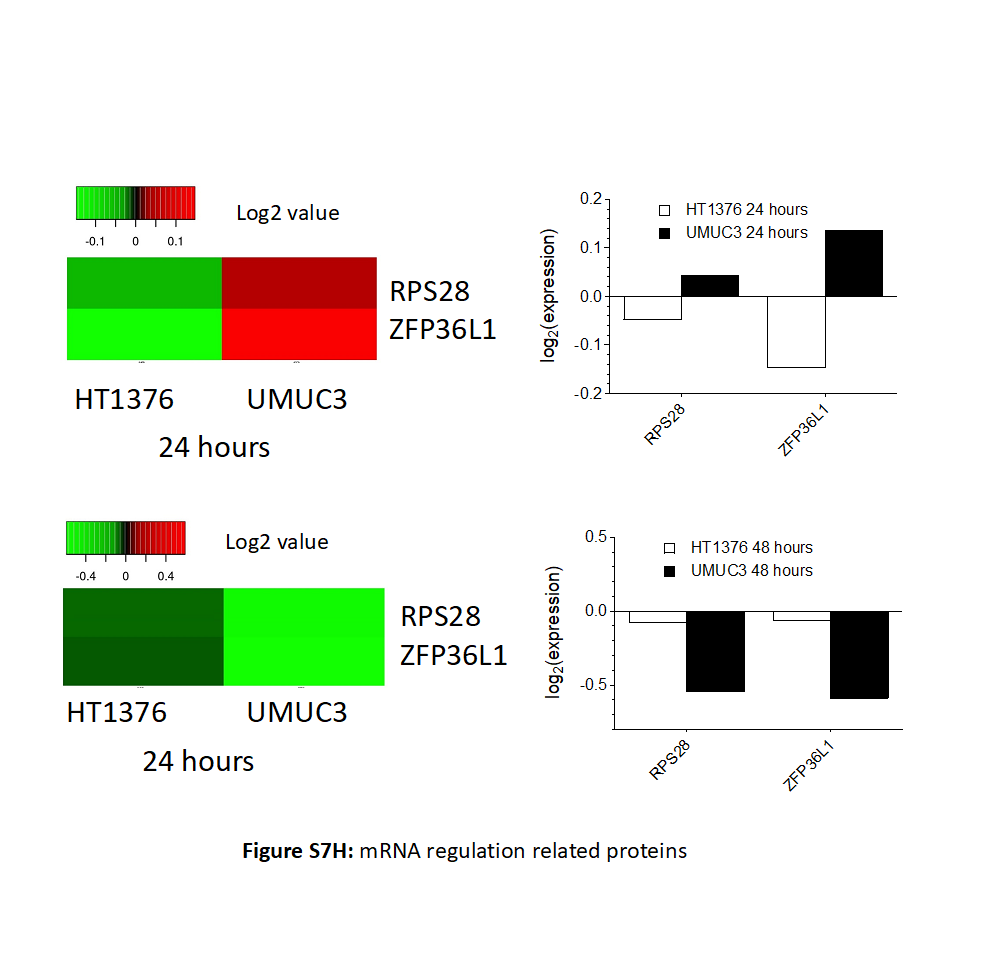


**Figure S7H.** mRNA regulation related proteins.


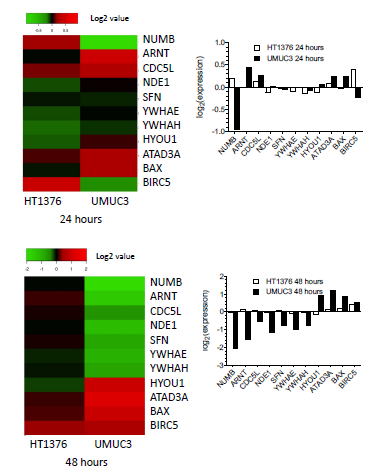


**Figure S7I.** Cell growth, proliferation and apoptosis related proteins.


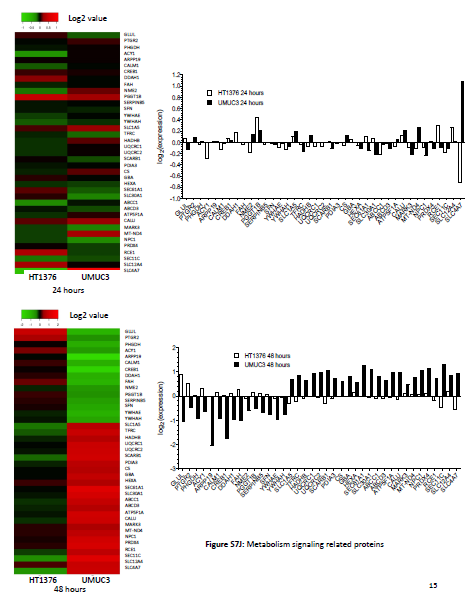


**Figure S7J.** Metabolism signaling related proteins.

**Figure S7K.** Blood metabolism related proteins.

**Figure S7L.** DNA metabolism and Damage repair related proteins.

**Figure S7M.** Transporter related proteins.

**Figure S7N.** Cell membrane, skeletal and golgi related proteins.

**Figure S7O.** Tissue structure and adhesion related proteins.

**Figure S7P.** Cell migration related proteins.

**Figure S7Q.** Cell vesicle traffic/formation signaling related proteins.

**Figure S7R.** Neuroscience-nerve system regulation signaling-related proteins.


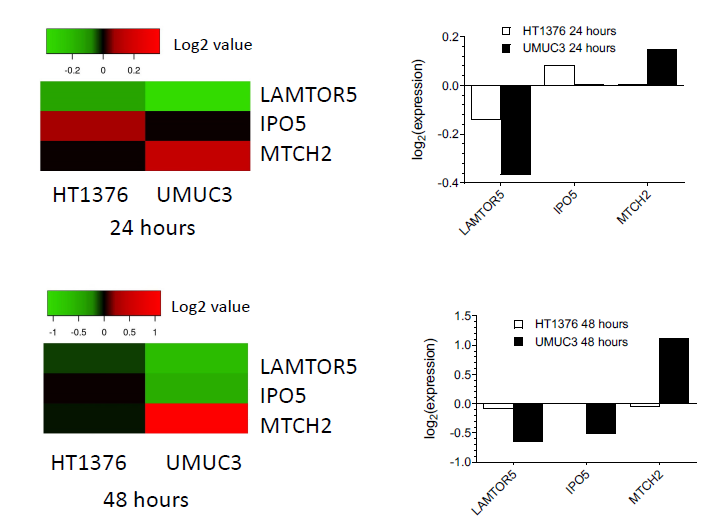


**Figure S7S.** Virus function related proteins.

**Figure S7T.** Cardiovascular and heart function related proteins.

**Figure S7U.** Eyes function related proteins.

**Figure S7V.** Protein folding correction relevant chaperonin family proteins.

**Figure S7W.** Nuclear pore complex (NPC) related proteins.

**Table S5.** Kras signaling pathway-relevant proteins (I)*.

| **Protein classification** | **Protein number and name in Table S3 with notes** |
| --- | --- |
| **Figure 8:** Ubiquitination (Ub) and deUb and proteasome related proteins | **31. CAND1** Cullin-associated NEDD8-dissociated protein 1 (Cullin-associated and neddylation-dissociated protein 1) (TBP-interacting protein of 120 kDa A) (TBP-interacting protein 120A) (p120 CAND1)  **49. PSMA1** Proteasome subunit alpha type-1 (EC 3.4.25.1) (30 kDa prosomal protein) (PROS-30) (Macropain subunit C2) (Multicatalytic endopeptidase complex subunit C2) (Proteasome component C2) (Proteasome nu chain)  **50. PSMA2** Proteasome subunit alpha type-3 (EC 3.4.25.1) (Macropain subunit C8) (Multicatalytic endopeptidase complex subunit C8) (Proteasome component C8)  **51. PMSA7** Proteasome subunit alpha type-7 (EC 3.4.25.1) (Proteasome subunit RC6-1) (Proteasome subunit XAPC7)  **52. PMSB1** Proteasome subunit beta type-1 (EC 3.4.25.1) (Macropain subunit C5) (Multicatalytic endopeptidase complex subunit C5) (Proteasome component C5) (Proteasome gamma chain)  **53. PMSB4** Proteasome subunit beta type-4 (EC 3.4.25.1) (26 kDa prosomal protein) (HsBPROS26) (PROS-26) (Macropain beta chain) (Multicatalytic endopeptidase complex beta chain) (Proteasome beta chain) (Proteasome chain 3) (HsN3)  **60. SFN** 14-3-3 protein sigma (Epithelial cell marker protein 1) (Stratifin)  **61. SKP2** S-phase kinase-associated protein 2 (Cyclin-A/CDK2-associated protein p45) (F-box protein Skp2) (F-box/LRR-repeat protein 1) (p45skp2)  **63. UBE2I** SUMO-conjugating enzyme UBC9 (EC 2.3.2.-) (RING-type E3 SUMO transferase UBC9) (SUMO-protein ligase) (Ubiquitin carrier protein 9) (Ubiquitin carrier protein I) (Ubiquitin-conjugating enzyme E2 I) (Ubiquitin-protein ligase I) (p18)  **64. UBE2N** Ubiquitin-conjugating enzyme E2 N (EC 2.3.2.23) (Bendless-like ubiquitin-conjugating enzyme) (E2 ubiquitin-conjugating enzyme N) (Ubc13) (UbcH13) (Ubiquitin carrier protein N) (Ubiquitin-protein ligase N)  **67. YWHAE** 14-3-3 protein epsilon (14-3-3E)  **68. YWHAH** 14-3-3 protein eta (Protein AS1) |
| **Figure S7A:** Cytokine and growth factor signaling related proteins | **9. MX1** Interferon-induced GTP-binding protein Mx1 (Interferon-induced protein p78) (IFI-78K) (Interferon-regulated resistance GTP-binding protein MxA) (Myxoma resistance  **15. GRB2** Growth factor receptor-bound protein 2 (Adapter protein GRB2) (Protein Ash) (SH2/SH3 adapter GRB2)  **39. IGF2BP2** Insulin-like growth factor 2 mRNA-binding protein 2 (IGF2 mRNA-binding protein 2) (IMP-2) (Hepatocellular carcinoma autoantigen p62) (IGF-II mRNA-binding protein 2) (VICKZ family member 2)  **41. IRF2** Interferon regulatory factor 2 (IRF-2)  **60. SFN** 14-3-3 protein sigma (Epithelial cell marker protein 1) (Stratifin)  **67. YWHAE** 14-3-3 protein epsilon (14-3-3E)  **68. YWHAH** 14-3-3 protein eta (Protein AS1) |
| **Figure S7B:** Immunotherapy related proteins and/or antigens |  |
| **Figure S7C:** Membrane receptor signaling related proteins | **2. ERRFI1** ERBB receptor feedback inhibitor 1 (Mitogen-inducible gene 6 protein) (MIG-6)  **5. RIN2** Ras and Rab interactor 2 (Ras association domain family 4) (Ras inhibitor JC265) (Ras interaction/interference protein 2)  **60. SFN** 14-3-3 protein sigma (Epithelial cell marker protein 1) (Stratifin)  **66. WDR44** WD repeat-containing protein 44 (Rabphilin-11)  **67. YWHAE** 14-3-3 protein epsilon (14-3-3E)  **68. YWHAH** 14-3-3 protein eta (Protein AS1) |
| **Figure S7D:** Cellular (non-membrane) signaling proteins | **6. STK3** Serine/threonine-protein kinase 3 (EC 2.7.11.1) (Mammalian STE20-like protein kinase 2) (MST-2) (STE20-like kinase MST2) (Serine/threonine-protein kinase Krs-1)  **20. MAP4K4** Mitogen-activated protein kinase kinase kinase kinase 4 (EC 2.7.11.1) (HPK/GCK-like kinase HGK) (MAPK/ERK kinase kinase kinase 4) (MEK kinase kinase 4) (MEKKK 4) (Nck-interacting kinase)  **60. SFN** 14-3-3 protein sigma (Epithelial cell marker protein 1) (Stratifin)  **67. YWHAE**14-3-3 protein epsilon (14-3-3E)  **68. YWHAH** 14-3-3 protein eta (Protein AS1) |
| **Figure S7E:** DNA and chromatin remodeling related proteins | **3. HELLS** Lymphoid-specific helicase (EC 3.6.4.-) (Proliferation-associated SNF2-like protein) (SWI/SNF2-related matrix-associated actin-dependent regulator of chromatin subfamily  **60. SFN** 14-3-3 protein sigma (Epithelial cell marker protein 1) (Stratifin)  **67. YWHAE** 14-3-3 protein epsilon (14-3-3E)  **68. YWHAH** 14-3-3 protein eta (Protein AS1) |
| **Figure S7F:** Transcription factor or co-factor related proteins | **4. APLP2** Amyloid-like protein 2 (APLP-2) (APPH) (Amyloid protein homolog) (CDEI box-binding protein) (CDEBP)  **7. CTNNB1** Catenin beta-1 (Beta-catenin)  **13. TCEA1** Transcription elongation factor A protein 1 (Transcription elongation factor S-II protein 1) (Transcription elongation factor TFIIS.o)  **16. CTNND1** Catenin delta-1 (Cadherin-associated Src substrate) (CAS) (p120 catenin) (p120(ctn)) (p120(cas))  **42. JUND** Transcription factor jun-D |
| **Figure S7G:** Gene expression and DNA damage related proteins | **26. ANP32A** Acidic leucine-rich nuclear phosphoprotein 32 family member A (Acidic nuclear phosphoprotein pp32) (pp32) (Leucine-rich acidic nuclear protein) (LANP) (Mapmodulin)  **60. SFN** 14-3-3 protein sigma (Epithelial cell marker protein 1) (Stratifin)  **67. YWHAE** 14-3-3 protein epsilon (14-3-3E)  **68. YWHAH** 14-3-3 protein eta (Protein AS1) |
| **Figure S7H:** mRNA regulation related proteins | **54. RPS28** 40S ribosomal protein S28 (Small ribosomal subunit protein eS28)  **69. ZFP36L1** mRNA decay activator protein ZFP36L1 (Butyrate response factor 1) (EGF-response factor 1) (ERF-1) (TPA-induced sequence 11b) (Zinc finger protein 36, C3H1 type-like 1) (ZFP36-like 1) |
| **Figure S7I:** Cell growth, proliferation and apoptosis related proteins | **8. NUMB** Protein numb homolog (h-Numb) (Protein S171)  **27. ARNT** Aryl hydrocarbon receptor nuclear translocator (ARNT protein) (Class E basic helix-loop-helix protein 2) (bHLHe2) (Dioxin receptor, nuclear translocator) (Hypoxia-inducible factor 1-beta) (HIF-1-beta) (HIF1-beta)  **32. CDC5L** Cell division cycle 5-like protein (Cdc5-like protein) (Pombe cdc5-related protein)  **43. NDE1** Nuclear distribution protein nudE homolog 1 (NudE)  **60. SFN** 14-3-3 protein sigma (Epithelial cell marker protein 1) (Stratifin)  **67. YWHAE** 14-3-3 protein epsilon (14-3-3E)  **68. YWHAH** 14-3-3 protein eta (Protein AS1) |
| **Figure S7J:** Metabolism signaling related proteins | **10. GLUL** Glutamine synthetase (GS) (EC 6.3.1.2) (Glutamate--ammonia ligase) (Palmitoyltransferase GLUL) (EC 2.3.1.225)  **11. PTGR2** Prostaglandin reductase 2 (PRG-2) (EC 1.3.1.48) (15-oxoprostaglandin 13-reductase) (Zinc-binding alcohol dehydrogenase domain-containing protein 1)  **17. PHGDH** D-3-phosphoglycerate dehydrogenase (3-PGDH) (EC 1.1.1.95) (2-oxoglutarate reductase) (EC 1.1.1.399) (Malate dehydrogenase) (EC 1.1.1.37)  **24. ACY1** Aminoacylase-1 (ACY-1) (EC 3.5.1.14) (N-acyl-L-amino-acid amidohydrolase)  **29. ARPP19** cAMP-regulated phosphoprotein 19 (ARPP-19)  **30. CALM1** Calmodulin-1  **35. CREB1** Cyclic AMP-responsive element-binding protein 1 (CREB-1) (cAMP-responsive element-binding protein 1)  **37. DDAH1** N(G),N(G)-dimethylarginine dimethylaminohydrolase 1 (DDAH-1) (Dimethylarginine dimethylaminohydrolase 1) (EC 3.5.3.18) (DDAHI) (Dimethylargininase-1)  **38. FAH** Fumarylacetoacetase (FAA) (EC 3.7.1.2) (Beta-diketonase) (Fumarylacetoacetate hydrolase)  **44. NME2** Nucleoside diphosphate kinase B (NDK B) (NDP kinase B) (EC 2.7.4.6) (C-myc purine-binding transcription factor PUF) (Histidine protein kinase NDKB) (EC 2.7.13.3) (nm23-H2)  **48. PGGT1B** Geranylgeranyl transferase type-1 subunit beta (EC 2.5.1.59) (Geranylgeranyl transferase type I subunit beta) (GGTase-I-beta) (Type I protein geranyl-geranyltransferase subunit beta)  **59. SERPINB5** Serpin B5 (Maspin) (Peptidase inhibitor 5) (PI-5)  **60. SFN** 14-3-3 protein sigma (Epithelial cell marker protein 1) (Stratifin)  **67. YWHAE** 14-3-3 protein epsilon (14-3-3E)  **68. YWHAH** 14-3-3 protein eta (Protein AS1) |
| **Figure S7K:** Blood metabolism related proteins | **58. SERBP1** Plasminogen activator inhibitor 1 RNA-binding protein (PAI1 RNA-binding protein 1) (PAI-RBP1) (SERPINE1 mRNA-binding protein 1)  **70. PLAT** Tissue-type plasminogen activator (t-PA) (t-plasminogen activator) (tPA) (EC 3.4.21.68) (Alteplase) (Reteplase) [Cleaved into: Tissue-type plasminogen activator chain A; Tissue-type plasminogen activator chain B] |
| **Figure S7L:** DNA metabolism and Damage repair related proteins | **45. PARP1** Poly [ADP-ribose] polymerase 1 (PARP-1) (EC 2.4.2.30) (ADP-ribosyltransferase diphtheria toxin-like 1) (ARTD1) (NAD(+) ADP-ribosyltransferase 1) (ADPRT 1) (Poly[ADP-ribose] synthase 1)  **60. SFN** 14-3-3 protein sigma (Epithelial cell marker protein 1) (Stratifin)  **67. YWHAE** 14-3-3 protein epsilon (14-3-3E)  **68. YWHAH** 14-3-3 protein eta (Protein AS1) |
| **Figure S7M:** Transporter related proteins |  |
| **Figure S7N:** Cell membrane, skeletal and golgi related proteins | **12. ADD3** Gamma-adducin (Adducin-like protein 70)  **22. ROCK1** Rho-associated protein kinase 1 (EC 2.7.11.1) (Renal carcinoma antigen NY-REN-35) (Rho-associated, coiled-coil-containing protein kinase 1) (Rho-associated, coiled-coil-containing protein kinase I) (ROCK-I) (p160 ROCK-1) (p160ROCK)  **25. ADD1** Alpha-adducin (Erythrocyte adducin subunit alpha)  **28. ARPC3** Actin-related protein 2/3 complex subunit 3 (Arp2/3 complex 21 kDa subunit) (p21-ARC)  **55. SEPT11** Septin-11  **56. SEPT7** Septin-7 (CDC10 protein homolog)  **57. SEPT8** Septin-8  **62. SORBS3** Vinexin (SH3-containing adapter molecule 1) (SCAM-1) (Sorbin and SH3 domain-containing protein 3)  **65. VIM** Vimentin |
| **Figure S7O:** Tissue structure and adhesion related proteins | **21. NCAM1** Neural cell adhesion molecule 1 (N-CAM-1) (NCAM-1) (CD antigen CD56)  **33. CDH2** Cadherin-2 (CDw325) (Neural cadherin) (N-cadherin) (CD antigen CD325)  **62. SORBS3** Vinexin (SH3-containing adapter molecule 1) (SCAM-1) (Sorbin and SH3 domain-containing protein 3) |
| **Figure S7P:** Cell migration related proteins | **34. CORO1C** Coronin-1C (Coronin-3) (hCRNN4)  **62. SORBS3** Vinexin (SH3-containing adapter molecule 1) (SCAM-1) (Sorbin and SH3 domain-containing protein 3) |
| **Figure S7Q:** Cell vesicle traffic/formation signaling related proteins | **14. EHBP1L1** EH domain-binding protein 1-like protein 1  **66. WDR44** WD repeat-containing protein 44 (Rabphilin-11) |
| **Figure S7R:** Neuroscience-nerve system regulation signaling-related proteins | **18. DLG1** Disks large homolog 1 (Synapse-associated protein 97) (SAP-97) (SAP97) (hDlg)  **36. DRN1** Drebrin (Developmentally-regulated brain protein) |
| **Figure S7S:** Virus function related proteins | **19. LAMTOR5** Ragulator complex protein LAMTOR5 (Hepatitis B virus X-interacting protein) (HBV X-interacting protein) (HBX-interacting protein) (Late endosomal/lysosomal adaptor and MAPK and MTOR activator 5)  **40. IPO5** Importin-5 (Imp5) (Importin subunit beta-3) (Karyopherin beta-3) (Ran-binding protein 5) (RanBP5) |
| **Figure S7T:** Cardiovascular and heart function related proteins | **23. SPTAN1** Spectrin alpha chain, non-erythrocytic 1 (Alpha-II spectrin) (Fodrin alpha chain) (Spectrin, non-erythroid alpha subunit)  **46. PDE4DIP** Myomegalin (Cardiomyopathy-associated protein 2) (Phosphodiesterase 4D-interacting protein)  **71. FSTL1** Follistatin-related protein 1 (Follistatin-like protein 1) |
| **Figure S7U:** Eyes function related proteins | **47. PDE6D** Retinal rod rhodopsin-sensitive cGMP 3',5'-cyclic phosphodiesterase subunit delta (GMP-PDE delta) (Protein p17) |
| **Figure S7V:** Protein folding correction relevant chaperonin family proteins |  |
| **Figure S7W:** Nuclear pore complex (NPC) related proteins |  |

* The proteins are classified in this table is from Table S3. These proteins are downregulated in UMUC3 cells but upregulated or no changes in HT1376 cells after FL118 treatment for 24h and 48h.

**Table S6.** Kras signaling pathway-relevant proteins (II)*.

| **Protein classification** | **Protein number and name in Table S4 with notes** |
| --- | --- |
| **Figure 8:** Ubiquitination (Ub) and deUb and proteasome related proteins | **2. SQSTM1** Sequestosome-1 (EBI3-associated protein of 60 kDa) (EBIAP) (p60) (Phosphotyrosine-independent ligand for the Lck SH2 domain of 62 kDa) (Ubiquitin-binding protein p62)  **4. ISG15** Ubiquitin-like protein ISG15 (Interferon-induced 15 kDa protein) (Interferon-induced 17 kDa protein) (IP17) (Ubiquitin cross-reactive protein) (hUCRP)  **39. TRAF6** TNF receptor-associated factor 6 (EC 2.3.2.27) (E3 ubiquitin-protein ligase TRAF6) (Interleukin-1 signal transducer) (RING finger protein 85) (RING-type E3 ubiquitin transferase TRAF6) |
| **Figure S7A:** Cytokine and growth factor signaling related proteins | **3. IRF9** Interferon regulatory factor 9 (IRF-9) (IFN-alpha-responsive transcription factor subunit) (ISGF3 p48 subunit) (Interferon-stimulated gene factor 3 gamma) (ISGF-3 gamma)  **7. IFIT1** Interferon-induced protein with tetratricopeptide repeats 1 (IFIT-1) (Interferon-induced 56 kDa protein) (IFI-56K) (P56)  **8. IFITM3** Interferon-induced transmembrane protein 3 (Dispanin subfamily A member 2b) (DSPA2b) (Interferon-inducible protein 1-8U)  **53. MET** Hepatocyte growth factor receptor (HGF receptor) (EC 2.7.10.1) (HGF/SF receptor) (Proto-oncogene c-Met) (Scatter factor receptor) (SF receptor) (Tyrosine-protein kinase Met) |
| **Figure S7B:** Immunotherapy related proteins and/or antigens | **9. CD274** Programmed cell death 1 ligand 1 (PD-L1) (PDCD1 ligand 1) (Programmed death ligand 1) (B7 homolog 1) (B7-H1) (CD antigen CD274)  **10. TGFB1** Transforming growth factor beta-1 proprotein [Cleaved into: Latency-associated peptide (LAP); Transforming growth factor beta-1 (TGF-beta-1)]  **18. HSPA5** Endoplasmic reticulum chaperone BiP (EC 3.6.4.10) (78 kDa glucose-regulated protein) (GRP-78) (Binding-immunoglobulin protein) (BiP) (Heat shock protein 70 family protein 5) (HSP70 family protein 5) (Heat shock protein family A member 5) (Immunoglobulin heavy chain-binding protein)  **34. MTCH2** Mitochondrial carrier homolog 2 (Met-induced mitochondrial protein): immune suppression and virus infection.  **46. BSG** Basigin (5F7) (Collagenase stimulatory factor) (Extracellular matrix metalloproteinase inducer) (**EMMPRIN**) (Leukocyte activation antigen M6) (OK blood group antigen) (Tumor cell-derived collagenase stimulatory factor) (TCSF) (CD antigen CD147): Ig related. As members of the immunoglobulin superfamily play fundamental roles in intercellular recognition involved in various immunologic phenomena, differentiation, and development, basigin is thought also to play a role in intercellular recognition and regulate several distinct functions, such as spermatogenesis, expression of the monocarboxylate transporter and the responsiveness of lymphocytes.  **65. THY1** Thy-1 membrane glycoprotein (CDw90) (Thy-1 antigen) (CD antigen CD90): May play a role in cell-cell or cell-ligand interactions during synaptogenesis and other events in the brain. |
| **Figure S7C:** Membrane receptor signaling related proteins | **11. EPHA2** Ephrin type-A receptor 2 (EC 2.7.10.1) (Epithelial cell kinase) (Tyrosine-protein kinase receptor ECK): implicated in mediating developmental events, particularly in the nervous system  **13. SDC4** Syndecan-4 (SYND4) (Amphiglycan) (Ryudocan core protein): interact with fibronectin on the cell surface, cytoskeletal and signaling proteins inside the cell to modulate the function of integrin in cell-matrix adhesion.  **28. GNB2** Guanine nucleotide-binding protein G(I)/G(S)/G(T) subunit beta-2 (G protein subunit beta-2) (Transducin beta chain 2)  **39. TRAF6** TNF receptor-associated factor 6 (EC 2.3.2.27) (E3 ubiquitin-protein ligase TRAF6) (Interleukin-1 signal transducer) (RING finger protein 85) (RING-type E3 ubiquitin transferase TRAF6)  **51. ITGA5** Integrin alpha-5 (CD49 antigen-like family member E) (Fibronectin receptor subunit alpha) (Integrin alpha-F) (VLA-5) (CD antigen CD49e) [Cleaved into: Integrin alpha-5 heavy chain; Integrin alpha-5 light chain]:  **61. RRAS2** Ras-related protein R-Ras2 (Ras-like protein TC21) (Teratocarcinoma oncogene)  **67. TNFRSF10A** Tumor necrosis factor receptor superfamily member 10A (Death receptor 4) (TNF-related apoptosis-inducing ligand receptor 1) (TRAIL receptor 1) (TRAIL-R1) (CD antigen CD261): Receptor for the cytotoxic ligand TNFSF10/TRAIL (PubMed:[26457518](https://www.uniprot.org/citations/26457518)). The adapter molecule FADD recruits caspase-8 to the activated receptor. The resulting death-inducing signaling complex (DISC) performs caspase-8 proteolytic activation which initiates the subsequent cascade of caspases (aspartate-specific cysteine proteases) mediating apoptosis (PubMed:[19090789](https://www.uniprot.org/citations/19090789)). Promotes the activation of NF-kappa-B (PubMed:[9430227](https://www.uniprot.org/citations/9430227)) |
| **Figure S7D:** Cellular (non-membrane) signaling proteins | **12. CDKN2A** Tumor suppressor ARF (Alternative reading frame) (ARF) (Cyclin-dependent kinase inhibitor 2A) (p14ARF)  **30. ARL6IP5** PRA1 family protein 3 (ADP-ribosylation factor-like protein 6-interacting protein 5) (ARL-6-interacting protein 5) (Aip-5) (Cytoskeleton-related vitamin A-responsive protein) (Dermal papilla-derived protein 11) (GTRAP3-18) (Glutamate transporter EAAC1-interacting protein) (JM5) (Prenylated Rab acceptor protein 2) (Protein JWa) (Putative MAPK-activating protein PM27) |
| **Figure S7E:** DNA and chromatin remodeling related proteins |  |
| **Figure S7F:** Transcription factor or co-factor related proteins | **5. JUNB** Transcription factor jun-B  **6. FOSL1**Fos-related antigen 1 (FRA-1)  **21. PHB** Prohibitin: Virus receptorl mitochondrial function and morphology, and transcriptional modulation (Co-factor)  **26. EEF1A1** Elongation factor 1-alpha 1 (EF-1-alpha-1) (Elongation factor Tu) (EF-Tu) (Eukaryotic elongation factor 1 A-1) (eEF1A-1) (Leukocyte receptor cluster member 7)  **29. NKRF** NF-kappa-B-repressing factor (NFkB-repressing factor) (Protein ITBA4) (Transcription factor NRF)  **60. RELA** Transcription factor p65 (Nuclear factor NF-kappa-B p65 subunit) (Nuclear factor of kappa light polypeptide gene enhancer in B-cells 3) |
| **Figure S7G:** Gene expression and DNA damage related proteins | **66. TMEM109** Transmembrane protein 109 (Mitsugumin-23) (Mg23): May mediate cellular response to DNA damage by protecting against ultraviolet C-induced cell death. Can form voltage-gated calcium and potassium channels in vitro (By similarity). |
| **Figure S7H:** mRNA regulation related proteins |  |
| **Figure S7I:** Cell growth, proliferation and apoptosis related proteins | **19. HYOU1** Hypoxia up-regulated protein 1 (150 kDa oxygen-regulated protein) (ORP-150) (170 kDa glucose-regulated protein) (GRP-170)  **42. ATAD3A** ATPase family AAA domain-containing protein 3A: antiapoptosis funciton  **44. BAX** Apoptosis regulator BAX (Bcl-2-like protein 4) (Bcl2-L-4)  **45. BIRC5** Baculoviral IAP repeat-containing protein 5 (Apoptosis inhibitor 4) (Apoptosis inhibitor survivin) |
| **Figure S7J:** Metabolism signaling related proteins | **14. SLC1AS** Neutral amino acid transporter B(0) (ATB(0)) (Baboon M7 virus receptor) (RD114/simian type D retrovirus receptor) (Sodium-dependent neutral amino acid transporter type 2) (Solute carrier family 1 member 5)  **15. TFRC** Transferrin receptor protein 1 (TR) (TfR) (TfR1) (Trfr) (T9) (p90) (CD antigen CD71) [Cleaved into: Transferrin receptor protein 1, serum form (sTfR)]  **17. HADHB** Trifunctional enzyme subunit beta, mitochondrial (TP-beta) [Includes: 3-ketoacyl-CoA thiolase (EC 2.3.1.16) (Acetyl-CoA acyltransferase) (Beta-ketothiolase)]  **23. UQCRC1** Cytochrome b-c1 complex subunit 1, mitochondrial (Complex III subunit 1) (Core protein I) (Ubiquinol-cytochrome-c reductase complex core protein 1)  **24. UQCRC2** Cytochrome b-c1 complex subunit 2, mitochondrial (Complex III subunit 2) (Core protein II) (Ubiquinol-cytochrome-c reductase complex core protein 2)  **25. SCARB1** Scavenger receptor class B member 1 (SRB1) (CD36 and LIMPII analogous 1) (CLA-1) (CD36 antigen-like 1) (Collagen type I receptor, thrombospondin receptor-like 1) (SR-BI) (CD antigen CD36)  **27. PDIA3** Protein disulfide-isomerase A3 (EC 5.3.4.1) (58 kDa glucose-regulated protein) (58 kDa microsomal protein) (p58) (Disulfide isomerase ER-60) (Endoplasmic reticulum  **31. CS** Citrate synthase, mitochondrial (EC 2.3.3.1) (Citrate (Si)-synthase)  **35. GBA** Glucosylceramidase (EC 3.2.1.45) (Acid beta-glucosidase) (Alglucerase) (Beta-glucocerebrosidase) (Beta-GC) (D-glucosyl-N-acylsphingosine glucohydrolase) (Imiglucerase)  **36. HEXA** Beta-hexosaminidase subunit alpha (EC 3.2.1.52) (Beta-N-acetylhexosaminidase subunit alpha) (Hexosaminidase subunit A) (N-acetyl-beta-glucosaminidase subunit alpha)  **37. SEC61A1** Protein transport protein Sec61 subunit alpha isoform 1 (Sec61 alpha-1)  **38. SLC30A1** Zinc transporter 1 (ZnT-1) (Solute carrier family 30 member 1)  **40. ABCC1** Multidrug resistance-associated protein 1 (ATP-binding cassette sub-family C member 1) (Leukotriene C(4) transporter) (LTC4 transporter)  **41. ABCD3** ATP-binding cassette sub-family D member 3 (70 kDa peroxisomal membrane protein) (PMP70)  **43. ATP5F1A** ATP synthase subunit alpha, mitochondrial (ATP synthase F1 subunit alpha)  **47. CALU** Calumenin (Crocalbin) (IEF SSP 9302): Involved in regulation of vitamin K-dependent carboxylation of multiple N-terminal glutamate residues.  **52. MARK3** MAP/microtubule affinity-regulating kinase 3 (EC 2.7.11.1) (C-TAK1) (cTAK1) (Cdc25C-associated protein kinase 1) (ELKL motif kinase 2) (EMK-2) (Protein kinase STK10) (Ser/Thr protein kinase PAR-1) (Par-1a) (Serine/threonine-protein kinase p78)  **54. MT-ND4** NADH-ubiquinone oxidoreductase chain 4 (EC 7.1.1.2) (NADH dehydrogenase subunit 4)  **55. NPC1** NPC intracellular cholesterol transporter 1 (Niemann-Pick C1 protein)  **58. PRDX4** Peroxiredoxin-4 (EC 1.11.1.15) (Antioxidant enzyme AOE372) (AOE37-2) (Peroxiredoxin IV) (Prx-IV) (Thioredoxin peroxidase AO372) (Thioredoxin-dependent peroxide reductase A0372)  **59. RCE1** CAAX prenyl protease 2 (EC 3.4.22.-) (Farnesylated proteins-converting enzyme 2) (FACE-2) (Prenyl protein-specific endoprotease 2) (RCE1 homolog) (hRCE1): Proteolytically removes the C-terminal three residues of farnesylated and geranylated proteins. Seems to be able to process K-Ras, N-Ras, H-Ras, RAP1B and G-gamma-1. Deubiquitination by USP17L2/USP17 negatively regulates the proteolytic activity toward Ras GTPases  **62. SEC11C** Signal peptidase complex catalytic subunit SEC11C (EC 3.4.21.89) (Microsomal signal peptidase 21 kDa subunit) (SPase 21 kDa subunit) (SEC11 homolog C) (SEC11-like protein 3) (SPC21): Component of the microsomal signal peptidase complex which removes signal peptides from nascent proteins as they are translocated into the lumen of the endoplasmic reticulum.  **64. SLC12A4** Solute carrier family 12 member 4 (Electroneutral potassium-chloride cotransporter 1) (Erythroid K-Cl cotransporter 1) (hKCC1)  **68. SLC4A7** Sodium bicarbonate cotransporter 3 (Electroneutral Na/HCO(3) cotransporter) (Sodium bicarbonate cotransporter 2) (Sodium bicarbonate cotransporter 2b) (Bicarbonate transporter) (Solute carrier family 4 member 7) |
| **Figure S7K:** Blood metabolism related proteins |  |
| **Figure S7L:** DNA metabolism and Damage repair related proteins |  |
| **Figure S7M:** Transporter related proteins | **14. SLC1A5** Neutral amino acid transporter B(0) (ATB(0)) (Baboon M7 virus receptor) (RD114/simian type D retrovirus receptor) (Sodium-dependent neutral amino acid transporter type 2) (Solute carrier family 1 member 5)  **30. ARL6IP5** PRA1 family protein 3 (ADP-ribosylation factor-like protein 6-interacting protein 5) (ARL-6-interacting protein 5) (Aip-5) (Cytoskeleton-related vitamin A-responsive protein) (Dermal papilla-derived protein 11) (GTRAP3-18) (Glutamate transporter EAAC1-interacting protein) (JM5) (Prenylated Rab acceptor protein 2) (Protein JWa) (Putative MAPK-activating protein PM27)  **37. SEC61A1** Protein transport protein Sec61 subunit alpha isoform 1 (Sec61 alpha-1)  **38. SLC30A1** Zinc transporter 1 (ZnT-1) (Solute carrier family 30 member 1)  **40. ABCC1** Multidrug resistance-associated protein 1 (ATP-binding cassette sub-family C member 1) (Leukotriene C(4) transporter) (LTC4 transporter)  **41. ABCD3** ATP-binding cassette sub-family D member 3 (70 kDa peroxisomal membrane protein) (PMP70)  **57. PANX1** Pannexin-1: transport of molecules of less than 1000 Da, or intercellular gap junctions. transport Ca^2+^, ATP, [inositol triphosphate](https://en.wikipedia.org/wiki/Inositol_trisphosphate) and other small molecules and can form hemichannels with greater ease than connexin subunit. Pannexin 1 and pannexin 2 underlie channel function in neurons and contribute to ischemic brain damage  **64. SLC12A4** Solute carrier family 12 member 4 (Electroneutral potassium-chloride cotransporter 1) (Erythroid K-Cl cotransporter 1) (hKCC1)  **68. SLC4A7** Sodium bicarbonate cotransporter 3 (Electroneutral Na/HCO(3) cotransporter) (Sodium bicarbonate cotransporter 2) (Sodium bicarbonate cotransporter 2b) (Bicarbonate transporter) (Solute carrier family 4 member 7) |
| **Figure S7N:** Cell membrane, skeletal and golgi related proteins | **16. GOLIM4** Golgi integral membrane protein 4 (Golgi integral membrane protein, cis) (GIMPc) (Golgi phosphoprotein 4) (Golgi-localized phosphoprotein of 130 kDa) (Golgi phosphoprotein of 130 kDa) |
| **Figure S7O:** Tissue structure and adhesion related proteins | **20. LAMB1** Laminin subunit beta-1 (Laminin B1 chain) (Laminin-1 subunit beta) (Laminin-10 subunit beta) (Laminin-12 subunit beta) (Laminin-2 subunit beta) (Laminin-6 subunit beta) (Laminin-8 subunit beta): Cerebral cortical development.  **33. LAMP1** Lysosome-associated membrane glycoprotein 1 (LAMP-1) (Lysosome-associated membrane protein 1) (CD107 antigen-like family member A) (CD antigen CD107a)  **50. FLOT2** Flotillin-2 (Epidermal surface antigen) (ESA) (Membrane component chromosome 17 surface marker 1): May act as a scaffolding protein within caveolar membranes, functionally participating in formation of caveolae or caveolae-like vesicles. May be involved in epidermal cell adhesion and epidermal structure and function.  **51. ITGA5** Integrin alpha-5 (CD49 antigen-like family member E) (Fibronectin receptor subunit alpha) (Integrin alpha-F) (VLA-5) (CD antigen CD49e) [Cleaved into: Integrin alpha-5 heavy chain; Integrin alpha-5 light chain]: adhesion and migration |
| **Figure S7P:** Cell migration related proteins | **33. LAMP1** Lysosome-associated membrane glycoprotein 1 (LAMP-1) (Lysosome-associated membrane protein 1) (CD107 antigen-like family member A) (CD antigen CD107a)  **51. IRGA5** Integrin alpha-5 (CD49 antigen-like family member E) (Fibronectin receptor subunit alpha) (Integrin alpha-F) (VLA-5) (CD antigen CD49e) [Cleaved into: Integrin alpha-5 heavy chain; Integrin alpha-5 light chain]: adhesion and migration |
| **Figure S7Q:** Cell vesicle traffic/formation signaling related proteins | **50. FLOT2** Flotillin-2 (Epidermal surface antigen) (ESA) (Membrane component chromosome 17 surface marker 1): May act as a scaffolding protein within caveolar membranes, functionally participating in formation of caveolae or caveolae-like vesicles. May be involved in epidermal cell adhesion and epidermal structure and function. |
| **Figure S7R:** Neuroscience-nerve system regulation signaling-related proteins |  |
| **Figure S7S:** Virus function related proteins | **34. MTCH2** Mitochondrial carrier homolog 2 (Met-induced mitochondrial protein): immune suppression and virus infection. |
| **Figure S7T:** Cardiovascular and heart function related proteins |  |
| **Figure S7U:** Eyes function related proteins | **22. STOM** Erythrocyte band 7 integral membrane protein (Protein 7.2b) (Stomatin): house-keeping protein with a possible structural role for this protein in the formation of these structures and/or the anchorage to the [actin](https://en.wikipedia.org/wiki/Actin) cytoskeleton |
| **Figure S7V:** Protein folding correction relevant chaperonin family proteins | **18. HSPA5** Endoplasmic reticulum chaperone BiP (EC 3.6.4.10) (78 kDa glucose-regulated protein) (GRP-78) (Binding-immunoglobulin protein) (BiP) (Heat shock protein 70 family protein 5) (HSP70 family protein 5) (Heat shock protein family A member 5) (Immunoglobulin heavy chain-binding protein)  **32. HSPD1** 60 kDa heat shock protein, mitochondrial (EC 3.6.4.9) (60 kDa chaperonin) (Chaperonin 60) (CPN60) (Heat shock protein 60) (HSP-60) (Hsp60) (HuCHA60) (Mitochondrial matrix protein P1) (P60 lymphocyte protein)  **48. DNAJA1** DnaJ homolog subfamily A member 1 (DnaJ protein homolog 2) (HSDJ) (Heat shock 40 kDa protein 4) (Heat shock protein J2) (HSJ-2) (Human DnaJ protein 2) (hDj-2): This gene encodes a member of the DnaJ family of proteins, which act as heat shock protein 70 cochaperones.  **49. DNAJA3** DnaJ homolog subfamily A member 3, mitochondrial (DnaJ protein Tid-1) (hTid-1) (Hepatocellular carcinoma-associated antigen 57) (Tumorous imaginal discs protein Tid56 homolog) |
| **Figure S7W:** Nuclear pore complex (NPC) related proteins | **56. NUP107** Nuclear pore complex protein Nup107 (107 kDa nucleoporin) (Nucleoporin Nup107): Plays a role in the nuclear pore complex (NPC) assembly and/or maintenance. Required for the assembly of peripheral proteins into the NPC. Involved in nephrogenesis  **63. SEC13** Protein SEC13 homolog (GATOR complex protein SEC13) (SEC13-like protein 1) (SEC13-related protein): Functions as a component of the nuclear pore complex (NPC). SEC13 is involved in the biogenesis of COPII-coated vesicles |

* The proteins are classified in this table is from Table S4. These proteins are upregulated in UMUC3 cells but downregulated or no changes in HT1376 cells after FL118 treatment for 24h and 48h.

| 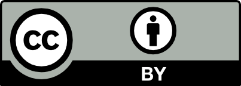 | © 2020 by the authors. Submitted for possible open access publication under the terms and conditions of the Creative Commons Attribution (CC BY) license (http://creativecommons.org/licenses/by/4.0/). |
| --- | --- |
